# Tube obstruction caused by blood clots following PreserFlo MicroShunt surgery

**DOI:** 10.1016/j.ajoc.2026.102560

**Published:** 2026-03-14

**Authors:** Kentaro Iwasaki, Masaru Inatani

**Affiliations:** Department of Ophthalmology, Faculty of Medical Sciences, University of Fukui, Fukui, Japan

**Keywords:** PreserFlo MicroShunt, Intraocular pressure, Glaucoma surgery, Tube obstruction, Blood clots, Hyphema, Minimally invasive, Anterior chamber washout

## Abstract

**Purpose:**

We describe a rare case of tube obstruction caused by blood clots following PreserFlo MicroShunt (PMS) surgery and its subsequent management.

**Principal results:**

A 64-year-old male patient with bilateral advanced exfoliation glaucoma underwent PMS surgery on the right eye. Tube obstruction was observed at 2 days postoperatively. Intraocular pressure (IOP) increased to 26 mmHg because of tube occlusion caused by blood clots at the tube tip. The occlusion was surgically released to free the lumen, and the IOP rapidly decreased to 7 mmHg.

**Major conclusions:**

Uncontrolled IOP caused by blood clots obstructing the tube post-PMS surgery can be resolved by surgical intervention without the need of tube reinsertion. Careful monitoring of the tube tip is required when hyphema occurs during the postoperative period of PMS surgery.

## 1 Introduction

The PreserFlo MicroShunt (PMS) (Santen Pharmaceutical Co. Ltd., Osaka, Japan) is a microinvasive filtration surgical device approved for use in Japan since February 2022. It is an aqueous humor drainage shunt designed to be implanted ab externo, creating a full-thickness fistula from the anterior chamber to the subconjunctival space and a filtering bleb. PMS surgery is a less invasive filtration surgery than trabeculectomy because it does not require scleral flap creation and iridectomy.^1^ Notably, PMS surgery is associated with fewer postoperative interventions and a lower incidence of hypotony compared with trabeculectomy. Both procedures are effective in lowering intraocular pressure (IOP); however, trabeculectomy tends to achieve lower mean IOPs at the possible cost of a higher risk of hypotony.^2^ PMS surgery may be performed as an alternative to trabeculectomy as the primary glaucoma surgery for patients with glaucoma and medically uncontrollable IOP.

Tube occlusion following PMS surgery is a relatively rare complication. Several cases of tube occlusion following PMS surgery caused by the fibrin, blood, iris, IOL capture, or vitreous have been documented.^3^, ^4^, ^5^, ^6^, ^7^, ^8^ We describe a rare case of tube obstruction caused by blood clots after PMS implantation and its successful surgical management without implant repositioning. We believe this case report will raise awareness of this complication and provide a reference for its management in similar situations.

## 2 Case report

A 64-year-old Japanese male presented with advanced bilateral exfoliation glaucoma. He had previously undergone cataract extraction and intraocular lens (IOL) implantation combined with a Kahook dual blade (KDB; New World Medical, Rancho Cucamonga, CA, USA) procedure in both eyes 2 years and 1 month earlier, as well as 2 micropulse transscleral cyclophotocoagulation treatments in the right eye—one performed 2 years ago and the other 2 months ago. Preoperatively, the best corrected visual acuity was 0.7 in the right eye and 0.3 in the left eye. Goldmann applanation tonometry showed an IOP of 28 mmHg in the right eye and 18 mmHg in the left eye using 5 different glaucoma medications (latanoprost 0.005% + carteolol hydrochloride 2% fixed combination, brimonidine tartrate 0.1% + brinzolamide 1% fixed combination, and ripasudil hydrochloride hydrate 0.4%). The elevated IOP in the right eye could not be controlled using glaucoma medications. The mean deviation in the right eye on the Humphrey visual field 24-2 SITA standard was −18.53 dB. The anterior segment of the right eye, especially the superior conjunctiva, were normal on slit-lamp examination, and no vitreous hemorrhage (VH) was observed. Standalone PMS implantation was scheduled for the right eye. The patient was not taking any anticoagulants or antiplatelet medications.

**Fig. 1:**
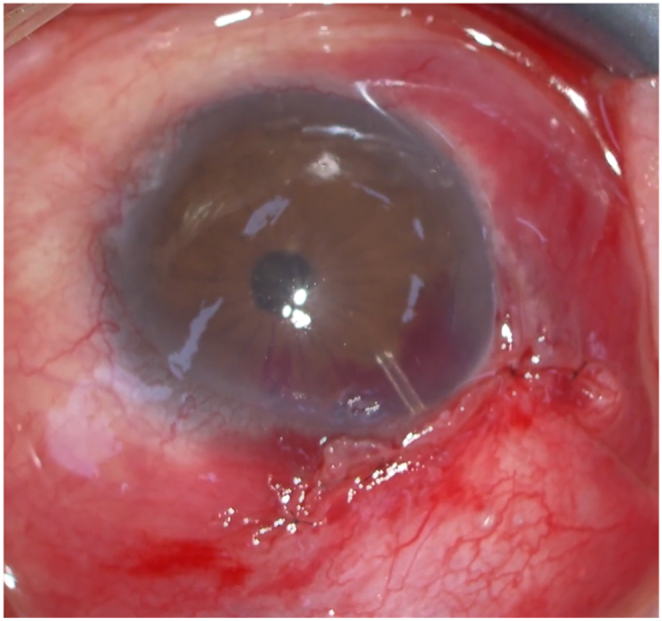
Intraoperative findings of PMS surgery (surgeon's view). A small hyphema was observed around the tube insertion site at the end of the surgery.

A corneal suture was placed on the superior cornea to control the globe. A 5-mm conjunctival incision was made along the limbus with posterior dissection to create a fornix-based conjunctival flap. Mitomycin C (0.4 mg/mL) was applied with sponges under the Tenon's capsule for 4 min, followed by irrigation with balanced salt solution (BSS) (100 mL). Limited cauterization was performed on the episcleral vessels at the PMS insertion site. The sclera was marked 3 mm posterior to the limbus, using the provided marking instrument. A scleral tunnel and pocket were created at this site using the provided double-step knife, and a small amount of bleeding into the anterior chamber was noted during this step. Subsequently, the PMS was inserted into the anterior chamber through the scleral tunnel, with its wings securely inserted into the scleral pocket. However, because the tube tip was close to the cornea during the first insertion, a second scleral tunnel and pocket were created. The PMS was then placed into the second tunnel in an appropriate position. The initial scleral tunnel was left untreated to allow spontaneous closure. Aqueous flow was confirmed by observing the drainage at the posterior PMS tip. The distal portion of the PMS was fixed to the scleral surface using 10-0 Nylon. The conjunctival flap was sutured watertight with 2 wing and mattress sutures at the limbus using 10-0 Nylon. At the end of the surgery, a small hyphema was observed (Fig. 1). The patient received postoperative topical medications with 0.5% moxifloxacin and 0.1% betamethasone sodium phosphate, both 3 times daily.

**Fig. 2:**
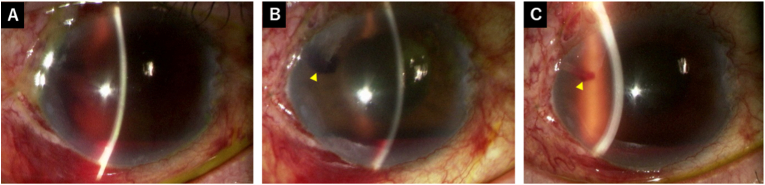
Postoperative findings after PMS surgery. (A) On postoperative day 1, the hyphema had a 3 mm height, and the tube could not be identified owing to bleeding. (B) On postoperative day 2, the tube was surrounded by blood clots (arrowhead). (C) On postoperative day 4, the hyphema had a 2 mm height, and tube tip obstruction caused by blood clots were suspected (arrowhead).

**Fig. 3:**
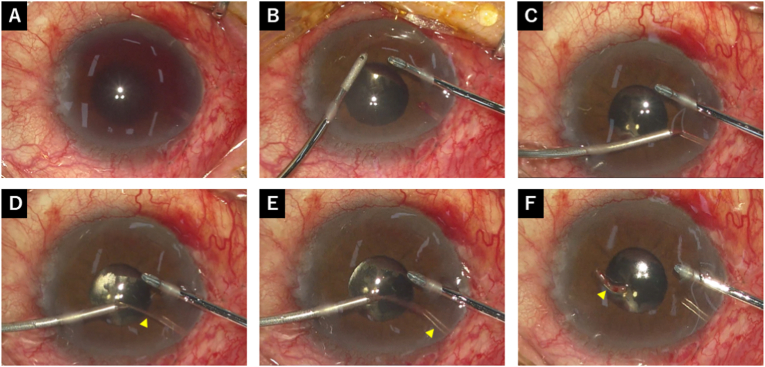
Intraoperative findings of anterior chamber washout (surgeon's view). (A) Intraoperative findings at the start of the surgery. (B) Anterior chamber washout was performed using bimanual irrigation and aspiration tips. (C) The foreign object was grabbed with forceps from the tube tip. (D) The foreign object (arrowhead) was pulled from the tube lumen with forceps. (E) Foreign objects were removed from the tube lumen, and the lumen was opened (arrowhead). (F) The removed foreign object was identified as a cylindrical fibrin clot (arrowhead).

On postoperative day 1, the IOP in the right eye was 17 mmHg, with a the hyphema of 3 mm height and flat bleb. The tube in the anterior chamber could not be identified owing to surrounding blood (Fig. 2A). On postoperative day 2, the IOP increased to 24 mmHg, and the tube was visible but surrounded by blood clots (Fig. 2B), indicating that the tube tip was blocked. Ocular massage was performed to lower the IOP and drain the bleeding from the tube, reducing the IOP to 13 mmHg. Ocular massage was applied 10 times on the side opposite to the tube. The bleb also changed from a flat bleb to a diffuse bleb after massage, and no aqueous leakage was observed. No glaucoma medications were started, and the postoperative topical regimen remained unchanged. On postoperative day 3, the IOP was 20 mmHg, and ocular massage converted the flat bleb into a diffuse bleb, lowering the IOP to 15 mmHg. On postoperative day 4, the IOP increased to 26 mmHg. The hyphema had decreased slightly; however, persistent tube tip obstruction was suspected because a clot was observed at the tube ostium (Fig. 2C), prompting an anterior chamber washout on the same day (Fig. 3). Two corneal side ports were created using a 20-gauge MVR-lance, and the washout was performed using bimanual irrigation and aspiration tips with a BSS. Anterior capsular forceps were used to remove cylindrical fibrin clots from the tube tip. Finally, the anterior chamber was filled with BSS, and the IOP was adjusted.

**Fig. 4:**
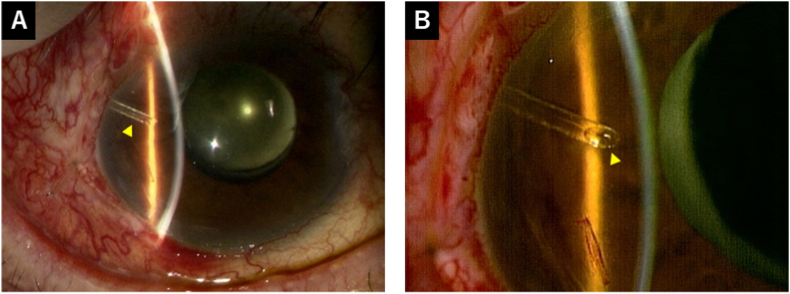
Postoperative findings 1 day after anterior chamber washout. (A) There was no hyphema and bleeding around the tube (arrowhead). (B) The tube tip was open (arrowhead).

**Fig. 5:**
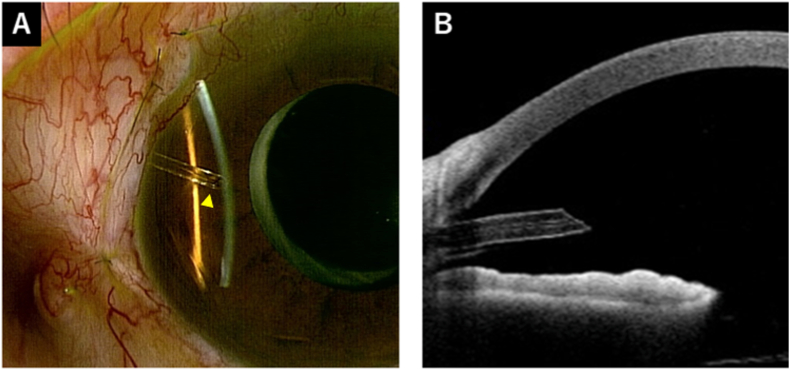
Postoperative findings at 7 weeks after anterior chamber washout. (A) The tube lumen was maintained unobstructed (arrowhead). (B) Anterior-segment optical coherence tomography showed an unobstructed tube.

One day after this secondary surgery, the IOP decreased to 7 mmHg, and the patient had a diffuse bleb. No hyphema was observed, and the tube tip was open (Fig. 4). VH was also confirmed. One week after the secondary surgery, the IOP was maintained at 7 mmHg without glaucoma medication. No hyphema was observed, and the tube lumen was maintained unobstructed. Three weeks post-secondary surgery, the IOP increased to 16 mmHg without glaucoma medication, likely due to a gradual decline in the filtration effect, as the diffuse bleb had also gradually become smaller. Seven weeks post-secondary surgery, the IOP was maintained at 16 mmHg without glaucoma medication, and the tube lumen was maintained unobstructed (Fig. 5). The VH showed a tendency to decrease.

## 3 Discussion

There are a few reports on tube obstruction after PMS implantation caused by the fibrin, blood, iris, IOL capture, and vitreous humor.^3^, ^4^, ^5^, ^6^, ^7^, ^8^ These occlusions were resolved using a yttrium-aluminum-garnet (YAG) laser, tissue plasminogen activator (tPA) injection, or surgical intervention. This was a rare case of tube obstruction caused by blood clots post-PMS surgery.

Previous reports have shown that postoperative hyphema occurs in 0%–20% of patients.^2^^,^^9^, ^10^, ^11^, ^12^, ^13^, ^14^ One study reported that postoperative hyphema occurred in 3 out of 70 cases, with 2 requiring anterior chamber washout and subsequent tPA injection for tube blockage.^9^ Postoperative intervention for hyphema is rarely required. Because the hyphema was small at the end of surgery (Fig. 1), we did not attempt to irrigate or aspirate it, nor did we increase the IOP with BSS to tamponade the bleeding. If these maneuvers had been performed, the amount of postoperative bleeding might have been reduced. The patient had previously undergone a goniotomy with a KDB, raising the possibility of reflux bleeding from the prior goniotomy site. However, the bleeding was identified at the tube insertion site (superotemporal), and as the KDB incision was located nasally, reflux bleeding from the goniotomy site was considered unlikely. In this case, anterior chamber washout and fibrin clot removal from the tube were necessary. Although it is uncertain whether early postoperative tPA injection would have prevented tube obstruction, tPA could also have been considered as a treatment option to dissolve the clot once it had formed. However, the use of tPA is off-label in Japan, limiting its availability.

Several glaucoma drainage devices are available for glaucoma surgery, and tube obstruction caused by blood or fibrin has been reported with many of these devices. Previous studies have described tube occlusion due to blood clots in valved implants such as the Ahmed glaucoma valve, as well as in Baerveldt glaucoma implants, and XEN gel stents.^15^, ^16^, ^17^, ^18^, ^19^ Therefore, blood-related tube obstruction is not unique to PMS and may occur in a variety of devices. The design characteristics of each implant, including inner diameter and tube length, may influence the likelihood of obstruction. The PMS tube (8.5 mm long, 70 μm inner diameter) is relatively long and narrow, potentially allowing blood to stagnate and form clots within the lumen. In contrast, the Ex-PRESS (2.64 mm length, 50 μm inner diameter) has a much shorter channel, whereas Baerveldt and Ahmed implants have longer but substantially wider tubes (350 μm and 305 μm, respectively). XEN (6 mm, 45 μm) has a similar lumen dimension and bleb-forming mechanism to PMS, and blood clot–related blockage has indeed been reported in XEN.^15^ These observations suggest that tube geometry may contribute to obstruction susceptibility, although it is not the sole determining factor. Furthermore, a previous study reported that in eyes with exfoliation glaucoma, increased protein concentration and viscosity of the aqueous humor, as well as exfoliative material, may predispose PMS tubes to blockage and poorer surgical outcomes.^20^

VH was not present preoperatively. VH occurred in this case; this is because eyes with exfoliation glaucoma are more prone to developing weak zonules,^21^^,^^22^ which may have allowed a large amount of hyphema to spread into the vitreous body. There were no underlying retinal or systemic diseases that could otherwise account for the VH.

Several reports have shown that tube occlusion after glaucoma drainage device surgery can be caused by the iris, blood clot, vitreous, or anterior capsule.^15^^,^^23^, ^24^, ^25^, ^26^, ^27^ For any type of tube occlusion, surgical procedures and YAG lasers are recommended. Surgical procedures to resolve tube occlusions include the use of forceps in the anterior chamber, anterior vitrectomy, and pars plana vitrectomy. In this case, we chose a surgical procedure to remove the hyphema and blood clots simultaneously. Consequently, the tube was clogged with cylindrical fibrin clot; therefore, surgery using forceps was appropriate in this case. Similarly, inner limiting membrane forceps was used to release the XEN tube occlusion caused by blood clots.^15^ In cases where the tube is surrounded by blood post-PMS surgery, early surgical intervention is advisable. In addition, tPA injection may be considered as another option for relieving tube obstruction by blood clot.^8^^,^^9^ However, because the use of tPA is off-label in some regions, including Japan, it may not be readily available in all clinical settings.

## 4 Conclusion

IOP elevation owing to tube obstruction caused by blood clots post-PMS surgery was resolved by surgical intervention without PMS reimplantation. Extra care is required regarding tube occlusion when hyphema occurs in the early postoperative period.

## CRediT authorship contribution statement

**Kentaro Iwasaki:** Writing – review & editing, Writing – original draft, Visualization, Validation, Supervision, Software, Resources, Project administration, Methodology, Investigation, Funding acquisition, Formal analysis, Data curation, Conceptualization. **Masaru Inatani:** Writing – review & editing, Supervision.

## Patient consent

The patient provided verbal informed consent for the publication of this case report and accompanying pictures.

## Statement of ethics

This case report was conducted in accordance with the Declaration of Helsinki and approved by the Institutional Review Board of the University of Fukui Hospital, Fukui, Japan.

## Authorship

All authors attest that they meet the current ICMJE criteria for authorship.

## Data statement

All data generated or analyzed during this study are included in this article. Further inquiries can be directed to the corresponding author.

## Declaration of generative AI in scientific writing

None.

## Funding sources

This study was partially supported by the Japan Society for the Promotion of Science (KAKENHI) (Grant number 22K16967).

## Declaration of competing interest

The authors declare that they have no known competing financial interests or personal relationships that could have appeared to influence the work reported in this paper.
